# P-2066. Impact of XXB 1.5 Monovalent Booster Vaccination on Mucosal and Systemic Immune Responses in Healthy Adults

**DOI:** 10.1093/ofid/ofae631.2222

**Published:** 2025-01-29

**Authors:** Nada Deraz, Michael C Payne, Ellen See, Vaishnavi Ragavapuram, Jürgen Bosch, Christopher King

**Affiliations:** University Hospitals - Rainbow Babies and Children, Beachwood, OH; Case Western Reserve University School of Medicine, University Heights, Ohio; Case Western Reserve University, Cleveland, Ohio; Case Western Reserve University, Cleveland, Ohio; Case Western Reserve University, Cleveland, Ohio; Case Western Reserve University, Cleveland, Ohio

## Abstract

**Background:**

The FDA recently approved the first monovalent XBB1.5 booster vaccine. However, the mechanism by which this vaccine stimulates mucosal and systemic immune responses has been understudied.

**Figure 1.**
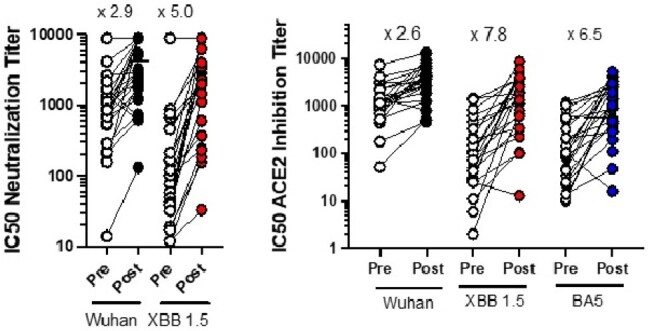
Impact of XBB 1.5 vaccination on viral neutralization titers using pseudo virus assay (left panel) and ACE2 inhibition assay (right panel). Each dot (white) represents a single serum sample tested in parallel with a post-vaccination sample (colored dot) for each of the 28 participants. The mean fold increase in neutralization titers pre- and post-vaccination are shown for Wuhan, XBB 1.5, and BA5 variants. All increases in neutralization titers are significant to P<0.0001 by employing Wilcoxon matched-pairs signed rank test.

**Methods:**

We examined 28 participants' serum and saliva one week before and two to three weeks after the XBB1.5 mRNA vaccination for neutralizing and binding antibodies to Wuhan and XBB1.5 S protein.

**Figure 2:**
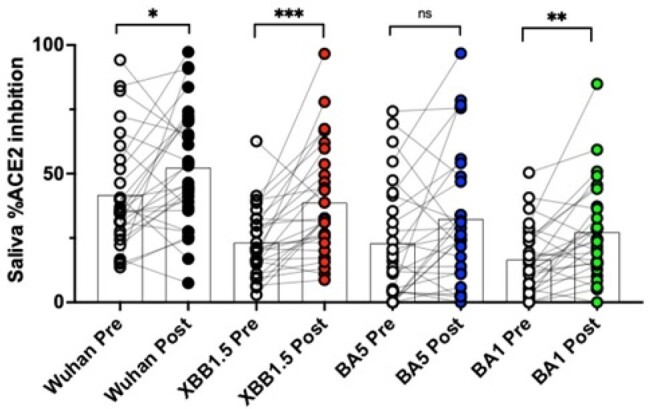
Inhibition of ACE2 binding to different S variants by saliva samples pre- and post-vaccination with XBB1.5 monovalent booster. Paired samples represent the percent ACE2 binding inhibition in participants before and after vaccination. Vaccination induced a 1.7-fold increase in ACE2 neutralization in saliva to XBB1.5 (p<0.0001) and 1.2-fold increase in ACE2 neutralization to Wuhan (p=0.03). Significance tested between pre and post-vaccination by Wilcoxon matched-paired sign rank test. *** p<0.001, **** p<0.0001.

**Results:**

We observed a 2.9 to 2.6-fold and 5.0 to 7.8-fold increase in Wuhan and XBB1.5 serum neutralization titers following mRNA vaccination using pseudovirus and ACE2 neutralization assays respectively, that closely correlated with a rise in binding antibodies to S protein. Vaccination induced a 1.7-fold increase in ACE2 neutralization in saliva to XBB1.5 (p< 0.0001) and 1.2-fold increase in ACE2 neutralization to Wuhan (p=0.03). This increase in neutralization Ab levels in saliva failed to correlate with the number and time of prior COVID-19 infections, vaccination status, type of vaccine, or concomitant rise in S-specific IgA or IgG in saliva or serum. Depletion of IgA or IgG in saliva following vaccination demonstrated that IgA was primarily responsible for the increase in ACE2 blocking activity (mean reduction in blocking activity with IgA depletion=68%, range, 58-81%) compared to IgG (mean reduction=27%, range 0-71%).

**Figure 3.**
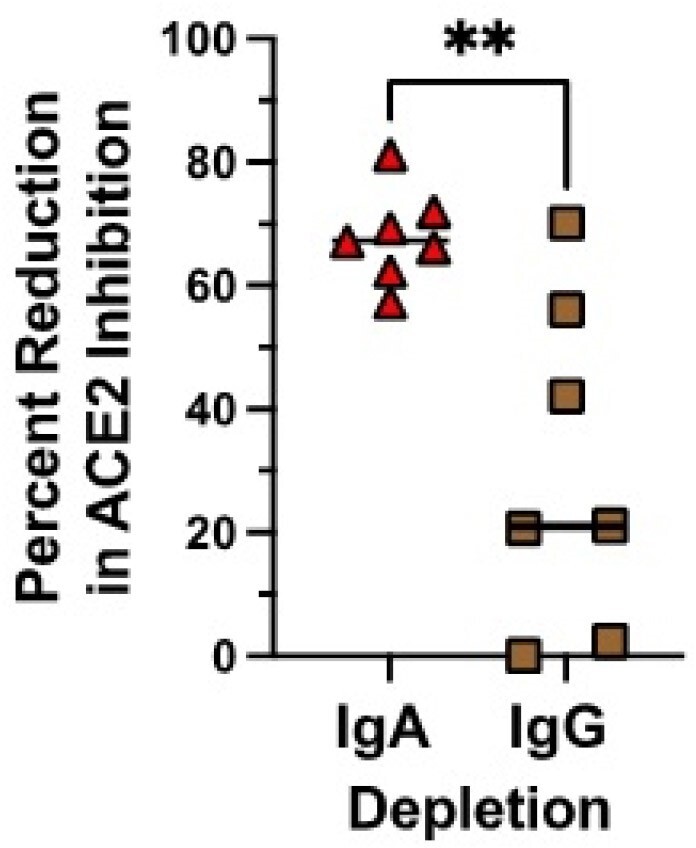
Role of IgA on percent reduction in ACE2 inhibition in saliva.

Depletion of IgA or IgG in saliva following vaccination demonstrated that IgA was primarily responsible for the increase in ACE2 blocking activity (mean reduction in blocking activity with IgA depletion=68%, range, 58-81%) compared to IgG (mean reduction=27%, range 0-71%). Statistical analysis done using unpaired t test.

**Conclusion:**

The XBB1.5 monovalent vaccine boosts systemic and mucosal viral neutralization antibody levels, primarily to the XBB1.5 variant. Most people have hybrid immunity to SARS-CoV2 now, so mRNA vaccines boost mucosal immune response better than observed earlier in the pandemic. Increased oral mucosal immunity may reduce the risk of COVID-19 progressing to pneumonia and more severe disease.

**Disclosures:**

All Authors: No reported disclosures

